# Disentangling genetic, plastic and social learning drivers of sex-specific foraging behaviour in Trinidadian guppies (*Poecilia reticulata*)

**DOI:** 10.1098/rspb.2023.2950

**Published:** 2024-03-13

**Authors:** Shayna R. Earl, Lauren E. Johnson, Elly Grant, Avika Kasubhai, Andrés López-Sepulcre, Yusan Yang, Swanne Gordon

**Affiliations:** ^1^ Department of Biology, Washington University in St. Louis, St. Louis, MO, USA; ^2^ Division of Biology and Biomedical Sciences, Washington University in St. Louis, St. Louis, MO, USA; ^3^ Department of Biology, University of Louisville, Louisville, KY, USA; ^4^ Department of Ecology and Evolution, Cornell University, Ithaca, NY, USA; ^5^ Department of Integrative Biology, University of South Florida, Tampa, FL, USA

**Keywords:** foraging behaviour, behavioural plasticity, social learning, gene-by-environment interaction

## Abstract

Evolutionary biologists have long been interested in parsing out the roles of genetics, plasticity and their interaction on adaptive trait divergence. Since males and females often have different ecological and reproductive roles, separating how their traits are shaped by interactions between their genes and environment is necessary and important. Here, we disentangle the sex-specific effects of genetic divergence, developmental plasticity, social learning and contextual plasticity on foraging behaviour in Trinidadian guppies (*Poecilia reticulata*) adapted to high- or low-predation habitats. We reared second-generation siblings from both predation regimes with or without predator chemical cues, and with adult conspecifics from either high- or low-predation habitats. We then quantified their foraging behaviour in water with and without predator chemical cues. We found that high-predation guppies forage more efficiently than low-predation guppies, but this behavioural difference is context-dependent and shaped by different mechanisms in males and females. Higher foraging efficiency in high-predation females is largely genetically determined, and to a smaller extent socially learned from conspecifics. However, in high-predation males, higher foraging efficiency is plastically induced by predator cues during development. Our study demonstrates sex-specific differences in genetic versus plastic responses in foraging behaviour, a trait of significance in organismal fitness and ecosystem dynamics.

## 1. Introduction

Behaviour is a complex trait that can rapidly adapt in response to changing environments [1], subsequently shaping an array of evolutionary and ecological processes [2–7]. While many behaviours are genetically determined [8–10], many are also plastic, wherein the same genotype produces multiple behavioural phenotypes in response to environmental stimuli. Plastic behavioural changes are important because they can outpace the rate at which genetic changes occur [11]. As environmental conditions continue to shift with increasing climate change, habitat alteration and anthropogenic influences, the capacity of species to rapidly adjust their behaviour is crucial for their continuing survival. Behaviours and their plasticity can also differ between sexes since they often have conflicting ecological and reproductive roles [10,12,13]. To elucidate the role of behaviour in adapting to novel or changing environments, assessing sex-specific genetic and plastic responses to environments is necessary.

Behavioural plasticity can take many forms. *Developmental plasticity* describes alternative trajectories into different phenotypes triggered by environmental stimuli during ontogeny, which are generally irreversible even after removal of the stimulus [11]. *Contextual plasticity,* on the other hand, refers to rapid changes in behaviour in response to stimuli in the immediate environment and can reverse after the stimulus is removed [11]. In many taxa, females generally display higher levels of developmental and contextual plasticity than males (reviewed in [14,15]). *Social learning* is a particularly interesting type of developmental plasticity in which the environmental stimuli are the behaviours of other individuals with whom the focal individuals interact (reviewed in [16–18]). Social learning can lead to the rapid spread of adaptive behaviours in a population, facilitating responses to environmental changes. It has also been shown to be sex-specific (reviewed in [19]); for example, Pogány *et al*. [20] found that male zebra finches learn more care behaviours than do females when reared with conspecifics.

Behavioural plasticity itself can be under selection. However, studies that examine the evolution of sex-specific plasticity in response to multiple environmental and social cues in genetically divergent populations are rare. Here, we disentangle the relative importance of genetic and plastic (including developmental, contextual and social learning) contributions as well as their gene-by-environment interactions (GxE) on sex-specific foraging behaviour between populations of Trinidadian guppies (*Poecilia reticulata*) adapted to high- and low-predation environments.

As populations diverge, they can experience variable environments that require different behavioural responses. Trinidadian guppies have repeatedly colonized two drastically different environments: downstream, high-predation (hereafter HP) sites and upstream, low-predation sites (hereafter LP; [21]). This has led to multiple instances of adaptive divergence in various behavioural traits, but also life history and morphology.

For example, it is well established that foraging behaviours differ between the sexes and have diverged in HP and LP environments. In the wild, HP guppies spend less time foraging, are more selective in their diet, and consume proportionally more invertebrates than LP guppies [22,23]. The differences in foraging behaviour between the two ecotypes of guppies have been previously shown to affect local ecosystem parameters ranging from algal and invertebrate abundance to decomposition rates [24]. Across the sexes, female guppies, especially those of larger body sizes, forage at higher rates because of their higher energetic needs compared with males [25–27]. To date, most studies examining sex-differences in foraging behaviour in guppies used wild-caught guppies, and thus little is known about the relative contribution of genetics and various forms of plasticity in the behavioural differences between HP and LP individuals (but see [28]).

In this study, we parsed how guppy foraging behaviour is shaped by genetic divergence between HP and LP habitats and plastic response to social and ecological cues. Specifically, we tested the interactions and relative contributions of: (i) population of origin (genetic divergence); (ii) predation cues during development (developmental plasticity); (iii) conspecific presence during development (social learning); and (iv) immediate predation cues (contextual plasticity) on foraging behaviour in male and female guppies. To do this, we reared second-generation HP and LP juveniles in water with or without predator cues, with either HP or LP conspecific adults, in a 2 × 2 × 2 full sibling factorial design. We then tested their foraging activities as adults in the presence and absence of predator cues. From prior studies and theory, we expect a combination of plastic and genetic responses. We also predict that the relative importance of plastic and genetic responses will differ between the sexes. Our experimental design also allows us to test whether behavioural plasticity itself has diverged between HP and LP populations and whether predation and social environment interact to shape foraging behaviour.

## 2. Methods

### (a) Study populations and rearing

We used second-generation (F_2_) fish reared under common garden conditions in the laboratory. In 2020, we collected guppies from a high-predation (HP) and low-predation (LP) population in the Aripo River on the island of Trinidad in the Southern Caribbean. The wild guppies were brought to the laboratory at Washington University in St. Louis, Missouri, treated for parasites, and then reared in group tanks separated by population. Offspring from the wild group tanks were then randomly paired and mated within populations. F_2_ offspring from these pairings were separated at birth and kept in individual 1.8 l tanks for two weeks. After two weeks, we split full siblings from the same breeding pair randomly into four treatments described below. In total, we had 282 F_2_ offspring (154 females and 128 males) from 24 mothers. Each breeding pair produced an average of 10.5 offspring (median = 9, minimum = 1, maximum = 32; electronic supplementary material, figure S1).

All experimental fish were kept in flow-through aquatic housing systems (Aquaneering Inc.) and maintained at 25°C under a 12 L : 12 D light cycle. Guppies were fed a quantified amount of brine shrimp (*Artemia* nauplii) twice a day.

### (b) Experimental design

F_2_ full siblings of HP and LP origins were split randomly into four rearing treatments, a 2 × 2 × 2 factorial design crossing the two population origins with two predator-rearing environments (the presence or the absence of olfactory predator cues) and two social environments (the presence of HP or LP conspecifics).

For predator environment treatments, we manipulated whether guppies developed in an environment mimicking HP or LP by rearing them in water with or without predator cues, following methods in Ghalambor *et al*. ([29]; *pred+* and *pred−* treatment, hereafter). In the *pred+* treatment, we connected a tank containing a pike cichlid (*Crenicichla alta*) to the flow-through water system of the guppies. The guppies could not see the predator, but they received chemical cues through the water. Each day the cichlid was fed two guppies, so the predator cue also included chemical signals from the predated guppies. In the *pred−* treatment, guppies were reared in separate flow-through racks that did not contain predator cues.

For the social environment treatments, we reared juveniles with conspecific adults from either HP or LP populations (HP or LP tutors, hereafter). We did this by adding two adult males and one adult female (the tutor fish) from either HP or LP population into the juvenile tank when the juveniles were two weeks old and large enough to avoid adult cannibalism.

Fish stayed in these treatments for approximately five weeks. Once the juveniles reached 45 days old the tutors were removed. The tutors were with the juvenile for a total of 31 days. The juveniles were assayed for foraging behaviours shortly after tutor removal, between 45 and 54 (45.69 ± 0.1) days old.

### (c) Foraging assay

For each focal juvenile fish, we measured their foraging behaviour twice, once in water with predator cues, and another in water without predator cues, in random order. Fish were therefore tested both in water that was identical to their rearing water, as well as the type of water they had not experienced before. This allowed us to test whether individuals showed context-dependent foraging behaviour (contextual plasticity) in response to immediate predation risk. The random order ensured that results were not biased by familiarity to water type or assay procedure. After tutors were removed, the focal fish were transferred to a 9.5 l tank filled with either *pred+* or *pred−* water and allowed to acclimatize overnight. To quantify foraging behaviour, we added 0.006 g of crushed Tetramin Tropical Flakes (Tetra Co.) to their tank. Fish were assessed between 10.00 and 22.00. We recorded the time to their first peck at the fish flakes (foraging latency, hereafter). We then recorded the total number of pecks for 3 min following the first peck (foraging rate, hereafter). Once the assay was completed, we transferred the fish to a new 9.5 l tank with the opposite water type, acclimatized them overnight, and repeated the above assay. If the fish did not peck for 10 min, we ended the assay and re-ran it the next day. If the fish did not feed after three assays of a given water type, they received a foraging rate of 0 and their latency score was considered right-censored.

### (d) Statistical analysis

All analyses were performed with R version 4.1.1 [30] in R Studio version 1.4.1717 [31].

To test the significance of genetics (population origin), developmental plasticity (predator treatment), social learning (social treatment) and contextual plasticity (assay water) on foraging behaviour, we fit mixed models on (i) foraging latency and (ii) foraging rate. Specifically, we analysed foraging latency using a Cox proportional hazards model using the function *coxme* from the *coxme* package [32]. Trials in which individuals did not attempt to forage were assigned a foraging latency of 600 s and considered as right-censored using the *surv* function in the *survival* package [21,33]. We analysed foraging rate using a negative binomial generalized linear mixed model (GLMM) (function *glmmTMB* from the package *glmmTMB*; [34]).

In both cases, we first fit a full model to the data on both sexes, where all four treatments and their two-way interactions were included as explanatory variables, as well as the interaction of sex with all other terms (to test for sex differences in genetic and plastic factors), leading to three-way interactions. Because three-way interactions are difficult to interpret, we next fit separate models for males and females, where all four factors and their two-way interactions were explanatory factors.

In all cases, to select the most parsimonious model, we used the *dredge* function from the package *MuMIn* [35], which selects the reduced model with the lowest Akaike information criterion (AIC) by comparing all possible models (see electronic supplementary material, tables S5–S8). When there were significant interactions, we performed conditional *post hoc* comparisons of the marginal means with the *emmeans* function from the package *emmeans* (see electronic supplementary material, tables S9–S20; [36]).

The results from the full models and model selection tables can be found in our electronic supplementary material, tables S1–S4. All selected models also included several interactions of sex with both main effects and interactions, justifying our decision to split the full model into sex-specific models.

For all four sex-specific models, we included population origin (HP, LP), predator treatment (*pred+*, *pred−*), social treatment (HP tutor, LP tutor), assay water (*pred+*, *pred−*) and all two-way interactions between the four variables as fixed effects. Refer to table 1 for biological explanations of each main effect and interaction. Individual identity was included as a random effect since each fish was repeatedly assayed in both *pred+* and *pred−* test water. Breeding pair (parents) identity was also included as a random effect since siblings were distributed across the experimental groups (electronic supplementary material, figure S1).

**Table 1.** Biological explanations of significant main effects and interactions. Grey boxes represent main effects. White boxes represent interactions. Interactions are either genotype-by-environment interactions (GxE) or environment-by-environment interactions (ExE).

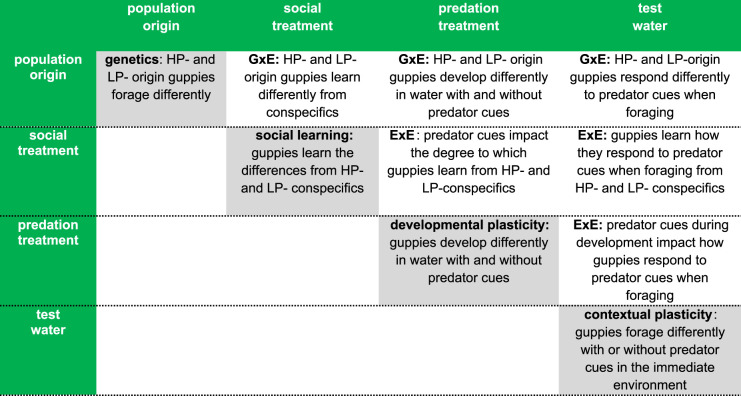

## 3. Results

The best-supported reduced models of foraging rate and foraging latency frequently included sex and its interactions with other mechanisms (electronic supplementary material, tables S2 and S4). In particular, the models of foraging rate included interactions of sex with population origin, predator treatment, tutor population, assay water and the interaction of predator treatment and assay water. The models of foraging latency included interactions of sex with population origin, predator treatment, tutor population, assay water, the interaction of predator treatment and tutor population, and the interaction of population origin and predator treatment. We next present the results of sex-specific models.

### (a) Patterns in female guppy foraging behaviour

**Figure 1.**
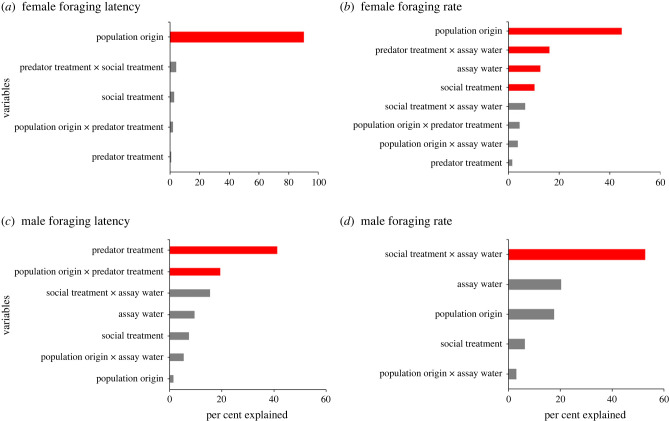
Relative importance of variables in a linear model predicting (*a*) female foraging latency, (*b*) female foraging rate, (*c*) male foraging latency, and (*d*) male foraging rate. The metrics of the main effects and interactions were scaled to sum to 100% for each behaviour and sex, respectively. Variables not listed were dropped based on AIC during model selection, and variables coloured red were statistically significant (*p* < 0.05).

Both measures of female foraging behaviour were shaped more by genetic divergence than developmental plasticity. Predator treatment only accounted for 0.7% and 1.5% of the explained variance in foraging latency and foraging rate, respectively, and social treatment accounted for just 2.8% and 10% (figure 1*a,b*; electronic supplementary material, tables S21 and S22). By contrast, population origin, the only significant predictor in the models, accounted for 90.3% and 44.8% of the explained variance in foraging latency and foraging rate (figure 1*a,b*; electronic supplementary material, tables S21 and S22). This suggests that genetic divergence is an important factor shaping female guppy foraging behaviour. An interaction of predator treatment and assay water also accounted for a substantial amount (16.2%) of variation in female foraging rate (figure 1*a*; electronic supplementary material, table S22), suggesting that females raised in *pred+* and *pred−* water responded differently to assay water.

**Figure 2.**
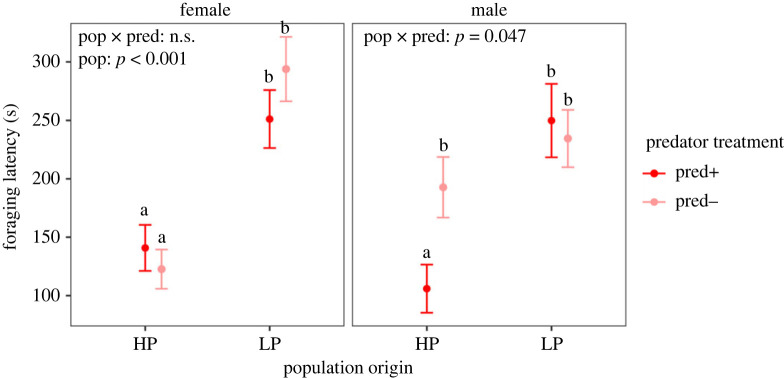
Foraging latency for female and male guppies separated by population origin (pop) and predator treatment (pred). Points represent mean and error bars represent standard error. Letters above indicate *post hoc* differences in marginal means. Points that do not share letters have marginal means that are statistically different with *p* < 0.05.

**Figure 3.**
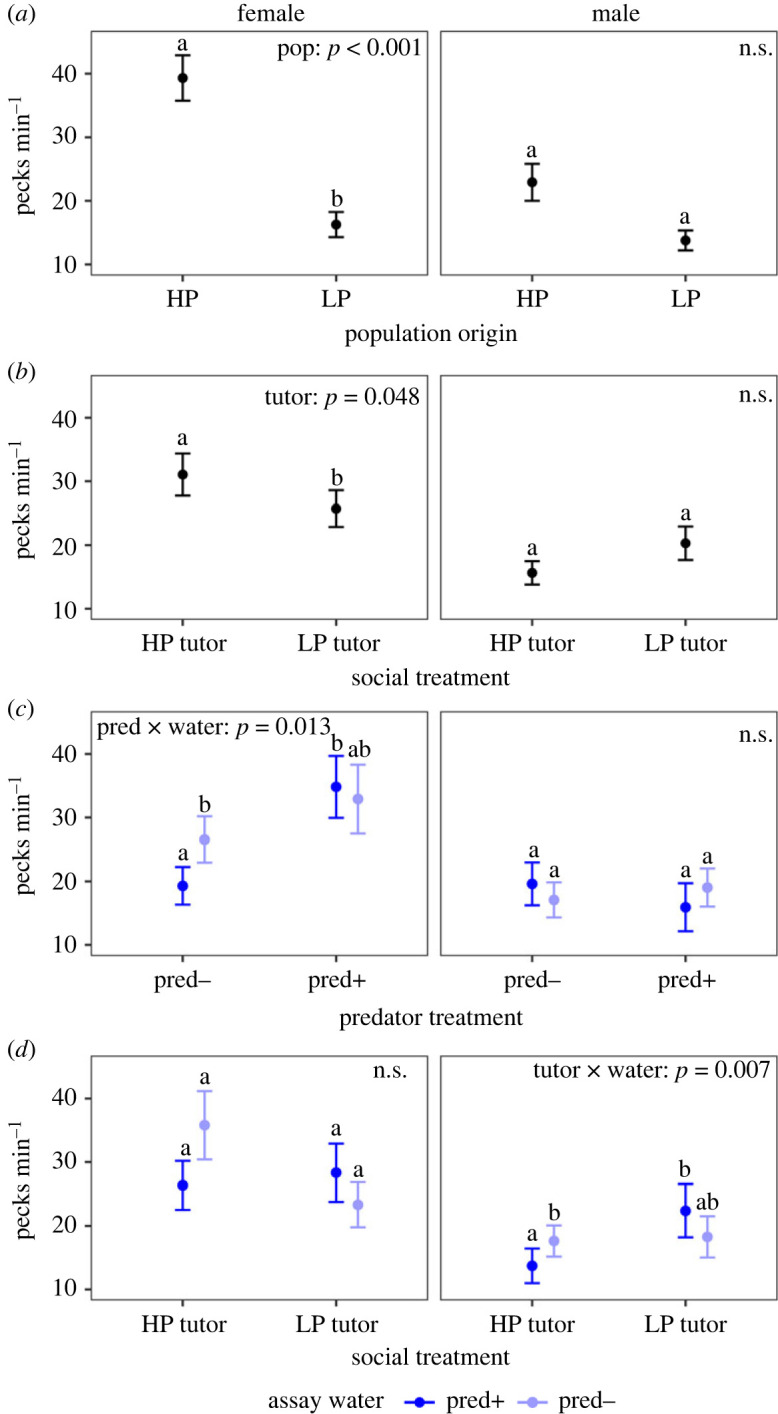
Foraging rate (pecks per minute) for female and male guppies separated by (*a*) population origin (pop), (*b*) social treatment (tutor), (*c*) predator treatment (pred) and assay water (water), (*d*) social treatment and assay water. Points represent mean and error bars represent standard error. Letters above indicate *post hoc* differences in marginal means. Points that do not share letters have marginal means that are statistically different with *p* < 0.05.

Specifically, our results showed that females from high-predation (HP) populations began foraging sooner than females from low-predation (LP) populations (figure 2; electronic supplementary material, table S21). Females from HP populations also pecked more frequently than females from LP populations (figure 3*a*; electronic supplementary material, table S22). Social treatment influenced foraging rate (figure 3*b*; electronic supplementary material, table S22) in that females reared with HP tutors pecked more frequently than females reared with LP tutors. There was also a significant interaction between predator treatment and assay water on female foraging rate (figure 3*c*; electronic supplementary material, table S22). Females raised in *pred−* water foraged more in *pred−* assay water than *pred+* assay water (Sidak: *b* ± s.e.: 0.60 ± 0.23, *z* = 2.67, *p* = 0.015; figure 3*c*; electronic supplementary material, table S20), while females raised in *pred+* water showed no difference in foraging rate when tested in the two assay water types (Sidak: *b* ± s.e. = −0.022 ± 0.25, *z* = −0.83, *p* = 0.64; figure 3*c*; electronic supplementary material, table S20).

### (b) Patterns in male guppy foraging behaviour

Unlike females, male foraging behaviour was shaped more by developmental plasticity and interactions between plasticity and genetic divergence than genetic divergence alone. Population origin accounted for 1.5% and 17.6% of the explained variance in foraging latency and foraging rate, respectively (figure 1*c,d*; electronic supplementary material, tables S23 and S24). In contrast, predator treatment explained 41% of the explained variance in foraging latency (figure 1*c*; electronic supplementary material, table S23), and an interaction of social treatment and assay water accounted for 52.7% of explained variance in foraging rates (figures 1*d*; electronic supplementary material, S24), suggesting that males raised with tutors from HP and LP populations responded differently to assay water. There was also an interaction of population origin and predator treatment for male foraging latency that accounted for 19.3% of explained variation (figure 1*c*; electronic supplementary material, figure S23), suggesting that HP and LP males responded differently to predator treatments.

Specifically, foraging latency in male guppies showed significant G×E interactions (figure 1*c*; electronic supplementary material, table S23). There were no differences in foraging latency between LP males raised in *pred−* or *pred+* water (Sidak: *b* ± s.e.: −0.01 ± 0.20, *z* = −0.04, *p* = 1.00; figure 2; electronic supplementary material, table S12). However, when HP males were raised in *pred+* water that mimicked their natural environment, they began foraging significantly sooner than those raised in *pred−* water (Sidak: *b* ± s.e.: −0.56 ± 0.19, *z* = −2.90, *p* = 0.008; electronic supplementary material, table S12; figure 2). Furthermore, foraging latency of HP males raised in *pred−* water did not differ significantly from either group of LP males (HP/pred*−* versus LP/pred*−* Sidak: *b* ± s.e.: 0.27 ± 0.18, *z* = 1.46, *p* = 0.61; HP/pred*−* versus LP/pred+ Sidak: *b* ± s.e.: 0.26 ± 0.20, *z* = 1.29, *p* = 0.73; electronic supplementary material, table S10; figure 2).

Foraging rate showed a significant interaction between social treatment and assay water (figure 1*d*; electronic supplementary material, table S24). Males raised with HP tutors foraged less when assayed in *pred+* water than when assayed in *pred−* water (Sidak: *b* ± s.e.: 0.62 ± 0.24, *z* = 2.62, *p* = 0.02; figure 2*d*; electronic supplementary material, table S16). By contrast, there were no significant effects of assay water on foraging rates in males raised with LP tutors (Sidak: *b* ± s.e.: −0.27 ± 0.23, *z* = −1.17, *p* = 0.42; figure 2*d*; electronic supplementary material, table S16).

## 4. Discussion

Our study has demonstrated that guppy foraging behaviour is shaped by a combination of genetics and plasticity (predation-induced contextual and developmental plasticity and social learning). In parsing out the importance of each, we found that these contributions differ drastically between the sexes. For females, genetic background is the most important determinant of foraging behaviour, with smaller contributions of social learning, and an interaction between developmental and contextual cues. Males show a more complex determination of foraging behaviours, with a GxE interaction (between population origin and predator treatment) determining foraging latency, and an interaction between social learning and contextual plasticity determining the rate of foraging. In quantifying the importance of these factors, our study contributes to the general understanding of a fundamental behavioural trait tightly related to both organismal fitness and the organism's impact on the environment [22].

### (a) Genetics

In many taxa, foraging behaviour is at least partly genetically based (e.g. bees [37] and birds [38]). In our study, population of origin was a strong predictor of female guppy foraging behaviour, suggesting genetic divergence between high- and low-predation habitats. This aligns with a previous report where female high-predation (HP) guppies begin foraging sooner than their low-predation (LP) conspecifics after the introduction of predator cues [39]. Higher foraging efficiency in HP females compared with LP females may be an adaptation to reduce total foraging time when they are vulnerable to predators [24]. Interestingly, population of origin was not the main determinant of male guppy foraging behaviour (figure 3). This aligns with the hypothesis that behaviour develops differently across sexes [10,12,13]. Wild adult males may spend less time foraging and, instead, invest more time courting and mating [26,40]. Therefore, sexual differences in genetic divergence in foraging behaviour may be because females are under stronger selection for higher foraging efficiency than males. Confirming this hypothesis will require further studies.

### (b) Predator-induced developmental plasticity

To understand the significance of predator-induced plasticity in adaptation, testing whether such developmental plasticity can evolve is necessary. Here we show that two populations that diverged relatively recently evolved different degrees of developmental plasticity in response to predator cues [41]. In foraging behaviour, HP males show plasticity in response to predator cues during development, but LP males do not. By contrast, neither HP nor LP females show predator cue-induced developmental plasticity. GxE interaction in male guppies suggests that developmental plasticity in response to predator cues was lost when HP guppies colonized and adapted to the upstream, LP habitats. Having developmentally plastic foraging latency may only be beneficial in the ancestral HP habitat where predation risk is more spatially or temporally variable. Confirming this adaptive function will require further experiments.

Interestingly, when considering the expressed phenotype in the wild, both male and female guppies achieve higher foraging efficiency (shorter starting latency and/or higher foraging rate) in HP environments. However, they do so via different mechanisms. Females diverged genetically on foraging behaviours, while males diverged in their capacity to be developmentally plastic. This sex-specific divergence suggests that responding quickly to temporal variation in predation risk may be more important for males than females. More broadly, this may be related to sexual differences in the trade-offs between foraging, reproductive behaviours and anti-predator responses [26,40,42–45].

### (c) Social learning

Social environment had a notable effect on the foraging rate of guppies. Females reared with HP tutors foraged at higher rates than those reared with LP tutors (figure 3*b*). Assuming that the HP tutors forage at a higher rate than LP tutors in our experiment, it suggests that female guppies learned to forage with a similar pattern as their social group. In comparison with genetic differences (population origin), social environment is a minor determinant of female foraging behaviour (figure 1*a,b*). The effect of social treatment was less conclusive in males. It is well established that female guppies are more sociable and rely more on public information than males [46,47]. This is probably due to male and female guppies having different physiological needs after reaching maturity: females have indeterminate growth and produce litters every month while males stop growing shortly after maturity and have lower energetic investments for reproduction [41]. As females have higher energy needs than males, being more receptive to social cues in learning when and where food is available probably confers a higher advantage for females. Broadly, this suggests that the evolution of behaviours in males and females may be decoupled and can be shaped by different proximate mechanisms.

Social learning of foraging behaviour has been documented in many animals (e.g. bees [48]; birds [49] and bats [50]), and more broadly, is fundamental to the development of various types of behaviour across taxa [17]. While the best-established model systems are in birds and mammals, there is increasing evidence of the importance of social learning in other organisms including fish [16], amphibians [3] and even non-colonial insects [51]. Our studies add to the increasing evidence that highlights the importance of social learning as a driver of adaptation and divergence.

### (d) Contextual plasticity

Immediate predation risk has some effects on the foraging behaviours of guppies, and this contextual plasticity can be shaped by the developmental environment. Our results demonstrated that female guppies developed a risk-sensitive foraging behaviour when they grew up with predator cues that signalled a high-risk environment. The interaction between social treatment and assay water is the largest determinant of male foraging rate. Males reared with HP tutors foraged less in *pred+* water compared with *pred−* water, while males reared with LP tutors foraged more in *pred+* water compared with *pred−* water. However, it is unlikely that this contextual plasticity resulted from social learning, because the individuals did not have the opportunity to see tutors foraging under different predator cue environments during rearing. Disentangling the proximate mechanism and adaptive significance of how the social environment influences contextual plasticity of foraging behaviour in male guppies would require further studies. The difference between male and female contextual plasticity may be because of the previously mentioned differences in energetic needs across sexes.

Contextual plasticity allows organisms to respond to the changing environment more quickly than evolution or developmental plasticity [1]. There are numerous examples of contextual behavioural plasticity in animals (reviewed in [52]). Guppies have shown contextual plasticity in response to predator cues in a variety of behaviours, including shoaling [53], courtship displays [54] and mate choice [55]. Here, we further show that the degree of contextual plasticity is shaped by experiences during development.

### (e) Sex-specific mechanisms shaping foraging behaviour

Overall, we propose several potential proximate and ultimate mechanisms that could lead to differences in foraging behaviour between the sexes. Sex differences in proximate mechanisms could be due to sex-specific hormonal levels and control leading to different reaction norms (see examples in lizards [56,57] and birds [58]), or differences in brain morphology which allow for varying levels of behavioural plasticity (e.g. in guppies [59] and birds [60]). The ultimate explanation of sex differences in genetic divergence and behavioural plasticity of foraging behaviour may be attributed to the differences in selective pressures acting on each sex. These different pressures can stem from sexual dimorphism in traits such as reproductive investment leading to different sex roles (see examples in birds [61] and frogs [62]). Natural and sexual selection can then shape the divergence of reaction norms in males and females separately by acting on various mechanistic levels, from hormone-controlled gene expression [63], sex chromosomes [64], to sex-specific brain development [59].

## 5. Conclusion

There is ample evidence of the influence of sex, genetics, developmental plasticity, social learning and activational plasticity on a variety of animal behaviours. Yet rarely are all five components evaluated jointly for their relative importance. We conducted a study that did exactly this and showed that foraging behaviour in guppies is shaped by complex interactions of these factors. Specifically, their relative importance varies significantly between males and females, with genetics primarily shaping female foraging and developmental plasticity playing a more crucial role in males. Our study assessed genetic, developmental, social and contextual influences on foraging behaviour, laying the groundwork for future research to pinpoint proximate and ultimate mechanisms shaping behavioural differences between sexes and populations.

Understanding the relative contribution of genetics, plasticity and learning to the expression of behaviour is crucial because it determines how organisms will respond to a novel environment, whether behaviour will evolve, and whether organisms can survive and thrive under a changing environment. Furthermore, foraging behaviour determines not only organismal fitness, but also their ecological impacts on the community and ecosystem [22,65]. To predict how animals adapt to a rapidly changing world, we must discern the relative importance of the different mechanisms that underpin their behavioural responses [66].

## Data Availability

Data and analysis codes can be accessed at: https://doi.org/10.5281/zenodo.10408080 [67]. Supplementary material is available online [68].
